# Characterization of rumen microbiome and immune genes expression of crossbred beef steers with divergent residual feed intake phenotypes

**DOI:** 10.1186/s12864-024-10150-3

**Published:** 2024-03-05

**Authors:** Godstime Taiwo, Olanrewaju B. Morenikeji, Modoluwamu Idowu, Taylor Sidney, Ajiboye Adekunle, Andres Pech Cervantes, Sunday Peters, Ibukun M. Ogunade

**Affiliations:** 1 Division of Animal and Nutritional Science, West Virginia University, 26505 Morgantown, WV USA; 2 Division of Biological and Health Sciences, University of Pittsburgh at Bradford, 300 Campus Drive, 16701 Bradford, PA USA; 3 Agricultural Research Station, Fort Valley State University, Fort Valley, GA USA; 4 Department of Animal Science, Berry College, Mount Berry, GA USA

**Keywords:** RFI, Rumen microbiota, Immunity

## Abstract

We investigated whole blood and hepatic mRNA expressions of immune genes and rumen microbiome of crossbred beef steers with divergent residual feed intake phenotype to identify relevant biological processes underpinning feed efficiency in beef cattle. Low-RFI beef steers (*n* = 20; RFI = − 1.83 kg/d) and high-RFI beef steers (*n* = 20; RFI = + 2.12 kg/d) were identified from a group of 108 growing crossbred beef steers (average BW = 282 ± 30.4 kg) fed a high-forage total mixed ration after a 70-d performance testing period. At the end of the 70-d testing period, liver biopsies and blood samples were collected for total RNA extraction and cDNA synthesis. Rumen fluid samples were also collected for analysis of the rumen microbial community. The mRNA expression of 84 genes related to innate and adaptive immunity was analyzed using pathway-focused PCR-based arrays. Differentially expressed genes were determined using *P*-value ≤ 0.05 and fold change (FC) ≥ 1.5 (in whole blood) or ≥ 2.0 (in the liver). Gene ontology analysis of the differentially expressed genes revealed that pathways related to pattern recognition receptor activity, positive regulation of phagocytosis, positive regulation of vitamin metabolic process, vascular endothelial growth factor production, positive regulation of epithelial tube formation and T-helper cell differentiation were significantly enriched (FDR < 0.05) in low-RFI steers. In the rumen, the relative abundance of *PeH15, Arthrobacter, Moryella, Weissella, and Muribaculaceae* was enriched in low-RFI steers, while *Methanobrevibacter, Bacteroidales_BS11_gut_group, Bacteroides and Clostridium_sensu_stricto_1* were reduced. In conclusion, our study found that low-RFI beef steers exhibit increased mRNA expression of genes related to immune cell functions in whole blood and liver tissues, specifically those involved in pathogen recognition and phagocytosis regulation. Additionally, these low-RFI steers showed differences in the relative abundance of some microbial taxa which may partially account for their improved feed efficiency compared to high-RFI steers.

**Supplementary Information:**

The online version contains supplementary material available at 10.1186/s12864-024-10150-3.

## Introduction

A vital role in animal development and health status is a cascade of events between the gut microbiomes and the host organism [1]. Studies have shown that the rumen microbiota have a profound impact on the health, performance, and immune system of the host [2, 3]. Rumen microbiome has been implicated as one of the major contributors to the variation in host feed efficiency in ruminants [4–6], due to their ability to produce the vast majority of energy precursors (sugar, lactate, H2) needed by the host animal coupled with other required micro-nutrients, such as all water-soluble vitamins [7, 8].

Over the past few decades, priority has been given to feed efficiency in the beef production system owing to an ever-increasing demand for animal products coupled with associated economic and environmental significance [9]. The most commonly used measure of feed efficiency in beef cattle is residual feed intake (RFI), which is the difference between observed feed intake and feed intake predicted from the animal’s maintenance and needs [10, 11]). In comparison to high-RFI cattle, low-RFI cattle consume less feed while maintaining normal growth levels. Several studies have sought to understand the metabolic processes underlying variation in RFI [12–14]. Some of the metabolic processes associated with RFI include energy metabolism, protein turnover, rumen microbial metabolism, and the immune system [15, 16]. In fact, rumen microbial activities and fermentation can influence ruminants’ performance, nutrient metabolism, and immune system [2, 3]. Innate and adaptive immune responses have high metabolic demands involving the repartitioning of nutrients when exposed to environmental stressors [17]. Due to the energy cost associated with immune system activation, immune competence is suggested to be one of the major physiological processes that contribute to variation in RFI in Angus beef cattle [18]. Despite these findings, differences in the metabolic demands of critical physiological processes in low- and high-RFI cattle such as immune responses and rumen microbiome have not been extensively studied. Furthermore, no studies have evaluated mRNA expression of innate and adaptive immunity-related genes and their associated regulatory pathways in beef steers’ blood and liver with divergent RFI phenotypes. Investigating rumen microbial community composition and diversity can provide insights into the mechanisms that regulate feed efficiency and help develop strategies to improve feed utilization and production efficiency in beef cattle. We hypothesized that selection for low- or high-RFI in beef cattle is associated with differences in hepatic and whole-blood immune gene expression and alteration in the relative abundance of rumen microbial taxa. Therefore, the objective of this study was to characterize the rumen microbiome and immune gene transcriptome of crossbred beef steers with divergent RFI phenotypes in order to gain insights into the mechanisms underlying differences in RFI.

## Materials and methods

### Animals and RFI determination

The use of animals in this experiment was approved by the Institutional Animal Care and Use Committees of West Virginia University (protocol number 1,608,003,693). This study involved feeding a high-forage total mixed ration (TMR; primarily consisting of corn silage; ground hay; and a ration balancing supplement; CP = 13.2%, NDF = 45.9% NDF, and NEg = 0.93 Mcal/kg) to 108 crossbred growing beef steers (average body weight of 282 ± 30.4 kg; age = 310 + 17 d) in a confinement dry lot equipped with GrowSafe intake nodes (GrowSafe Systems Ltd., Airdrie, Alberta, Canada) for a total of 70 d. Steers had unrestricted access to the experimental diet and water. Individual steer’s feed intake and daily BW were measured with GrowSafe automated feed intake and In-Pen Weighing Positions (IPW Positions, Vytelle LLC), respectively [19, 20]. The IPW Positions measured the partial BW of the animals by weighing the front end every second the animals stayed on the scale while drinking. Approximately 702 ± 102 daily BW data points (after filtering outliers) per animal were generated and were regressed on time using simple linear regression to calculate beginning BW, mid-test BW, and average daily gain (ADG). Average daily gain (ADG) and metabolic mid-test BW (mid-test BW^0.75^; MMTW) were regressed against daily DM intake. The following equation was used to calculate RFI, which is the difference between the predicted value from the regression and the actual measured value: Y = β_0_ + β_1_ × _1_ + β_2_ × _2_ + ε, where Y is the predicted DMI (kg/d), β_0_ is the regression intercept, β_1_ and β_2_ are the partial regression coefficients, X_1_ is the MMTW (kg), X_2_ is the ADG (kg/d), and ε is the error term [21]. The RFI coefficient (kg/d) for each beef steer was then calculated as the difference between the actual and predicted DMI. After calculating RFI values for all animals, the beef steers with the lowest RFI (*n* = 20; referred to as low-RFI) and the ones with the highest RFI (*n* = 20; referred to as high-RFI) were identified as the most and least efficient, respectively.

### Blood, rumen fluid and liver biopsy collection

On day 70, 10 mL of blood was collected from each animal prior to morning feeding (after overnight feed withdrawal) and placed into tubes containing sodium heparin. Subsequently, subsamples of 500 µL each were promptly transferred into RNA-protect tubes (Cat. No. 76,554; Qiagen) that contains a reagent capable of lysing blood cells and stabilizing intracellular RNA. The samples were stored at -80 °C until they were later analyzed. Liver biopsy procedure was also carried out on d 70 as described by Swanson et al., 2000. After excising the skin, liver tissue was extracted using a 14-gauge biopsy needle (TruCore-II Automatic Biopsy Instrument: Angiotech, Lausanne, Switzerland) and during a single puncture, approximately 1 g of liver samples were obtained from each of the beef steers. Liver samples were immediately stored in RNAprotect tissue tubes (Cat No: 76,163; Qiagen, Germantown, MD), and were immediately stored at -80 °C until they were analyzed. On the same day (day 70), rumen fluid samples were collected 4 h after feeding as described by [22]. Briefly, an orally administered stomach tube connected to a vacuum pump (Ruminator; **profs-products.com**, Wittibreut, Bayern, Germany) was used. To reduce saliva contamination, the first 150 mL of the collected rumen fluid samples were discarded. Subsequently, approximately 200 mL of rumen fluid was collected and promptly stored at -80 °C until later analysis. Liver biopsy procedure was also carried out on d 70 as described by Swanson et al., 2000. After excising the skin, liver tissue was extracted using a 14-gauge biopsy needle (TruCore-II Automatic Biopsy Instrument: Angiotech, Lausanne, Switzerland) and during a single puncture, approximately 1 g of liver samples were obtained from each of the beef steers. Liver samples were immediately stored in RNAprotect tissue tubes (Cat No: 76,163; Qiagen, Germantown, MD) containing RNAprotect tissue reagent that immediately stabilizes RNA in tissue samples to preserve the gene expression profile, and thereafter stored at -80 °C until later analysis.

### DNA extraction, 16 S rRNA sequencing and sequence analysis

The thawed rumen fluid samples were centrifuged at 15,000 × g, and the resulting pellets were used for DNA extraction using a PowerSoil DNA isolation kit (MO BIO Laboratories Inc., Carlsbad, CA). The concentration and purity of the extracted DNA were assessed using a NanoDrop 2000 UV-vis Spectrophotometer (Thermo Scientific, Wilmington, DE, United States). The integrity of DNA was tested using 0.7% agarose gel electrophoresis (Axygen Biosciences, Union City, CA, United States). The DNA samples were prepared for PCR using Qiagen QIAseq phased primers that target the V3/V4 regions of the 16 S gene following the manufacturer’s instruction (Qiagen; catalog number: 333,845). The forward and reverse primer sequences are CCTACGGGNGGCWGCAG and GACTACHVGGGTATCTAATCC respectively. Following the PCR amplicon cleaning, the samples were sequenced on a v3 MiSeq 600-cycle flowcell to generate 2 × 276 bp PE reads. Quality control and adapter trimming of the raw sequence files were performed using Illumina binary base call Convert v4.0. The fastq files generated were imported into Qiime2 [23] for subsequent analysis. Primer sequences were removed using Qiime2’s cutadapt plugin. The sequences were denoised using Qiime2’s dada2 plugin. Denoised sequences were annotated as operational taxonomic units (OTUs) using the Silva database with a sequence similarity threshold of 97% [24]. Analyses of the OTU data were performed using MicrobiomeAnalyst platform (microbiomeanalyst.ca; [25]). First, cumulative-sum scaling and log2 transformation of the OTU abundance data were performed for normalization. Rarefaction curves, alpha diversity (Chao1 index) and beta diversity (Bray-Curtis distance matrix based on principal coordinates analysis (PCoA)) were generated. Differentially abundant taxa at the phylum and genus levels were analyzed and determined using the linear discriminant analysis (LDA) effect size method (LEfSe) based on Kruskal–Wallis test of α ≤ 0.05 and logarithmic LDA score cut-off of 2.0.

### RNA extraction, cDNA synthesis and immune gene expression

Total RNA was isolated from the liver and whole blood samples using RNeasy Micro Kit (Cat No: 74,004; Qiagen) and RNeasy Protect Animal Blood kit (Cat. No. 73,224; Qiagen). RNA concentration was measured using a NanoDrop One C spectrophotometer (Thermo Fisher Scientific, Waltham, MA, USA). RNA samples were screened for quality using the Agilent 2100 Bioanalyzer (Agilent Technologies; Santa Clara, CA), ensuring that all samples exhibited RNA integrity numbers exceeding 8.0. Additionally, only samples with A260:A280 ratios falling within the range of 1.8 to 2.0 were selected for cDNA synthesis, accomplished using the RT^2^ First Strand Kit (Cat. No. 330,401; Qiagen). The expression of 84 genes associated with innate and adaptive immune responses was analyzed using the cow RT2 Profiler PCR Array (PABT-052ZA; Qiagen) according to the manufacturer’s instructions. Description of the RT2 Profiler PCR Array has been published in our previous study [26]. The real-time PCR analysis was carried out on a QuantStudio 5 Block Real-Time PCR System (Applied Biosystems, Foster City, CA) using the following cycling conditions: 95 °C for 10 min, 40 cycles of denaturation at 95 °C for 15 s and 60 °C 1 min [27, 28].

### Gene expression, gene ontology and pathway analyses

The Qiagen platform-web GeneGlobe (https://www.qiagen.com) was utilized for analyzing the immune gene expression data. Relative quantification of the gene expression was determined using the comparative cycle threshold (Ct) method [29]. To determine the differential mRNA expression between the low- and high-RFI beef steers, the delta-delta-Ct (ΔΔCt) method was employed, with normalization of the raw data using the geometric mean of the five housekeeping genes, as described by [29]. The mRNA expression with *P*-value ≤ 0.05 and fold change (FC) ≥ 1.5 (in blood) and ≥ 2.0 (in liver) were considered to be differentially expressed. We applied different FC thresholds between the blood and liver because the liver, being a complex organ with diverse functions including metabolism and immunity, often exhibits higher baseline gene expression levels and variability compared to the whole blood. Gene ontology (GO) terms and pathways analyses of differentially expressed genes were performed using a web-based geneontology software (http://www.geneontology.org) as described by [30]. Significantly enriched pathways among the differentially expressed genes were catalogued using false discovery rate-adjusted *P*-values (FDR; [31] ) ≤ 0.05.

## Results

### Growth performance of the low and high-RFI beef steers

The RFI values of low- and high-RFI steers were − 1.83 kg/d and + 2.12 kg/d (*P* = 0.001, SE = 0.41), respectively. The initial BW, final BW, and ADG were not different between the two groups (*P* > 0.05); however, low-RFI steers had lower (*P* = 0.01) DMI and feed:gain ratio compared to the high-RFI steers (Table 1).

**Table 1:** Growth performance of the low and high-RFI beef steers

Parameters	^1^Low-RFI	^2^High-RFI	SE	*P*-value
RFI, Kg/d	-1.83	2.12	0.41	0.01
Initial BW, Kg	313	345	10.18	0.14
Final BW, Kg	430	467	12.92	0.19
ADG, Kg/d	1.68	1.74	0.05	0.60
DMI, Kg/d	9.02	11.5	0.33	0.01
F:G	2.38	2.98	0.09	0.01

^1^Low-RFI = feed-efficient beef steers, ^2^High-RFI = feed inefficient beef steers. ADG, average daily gain; DMI, dry matter intake; BW, body weight; F: G, feed: gain ratio; SE, standard error of mean

### Sequencing results and rumen microbial community

**Fig. 1:**
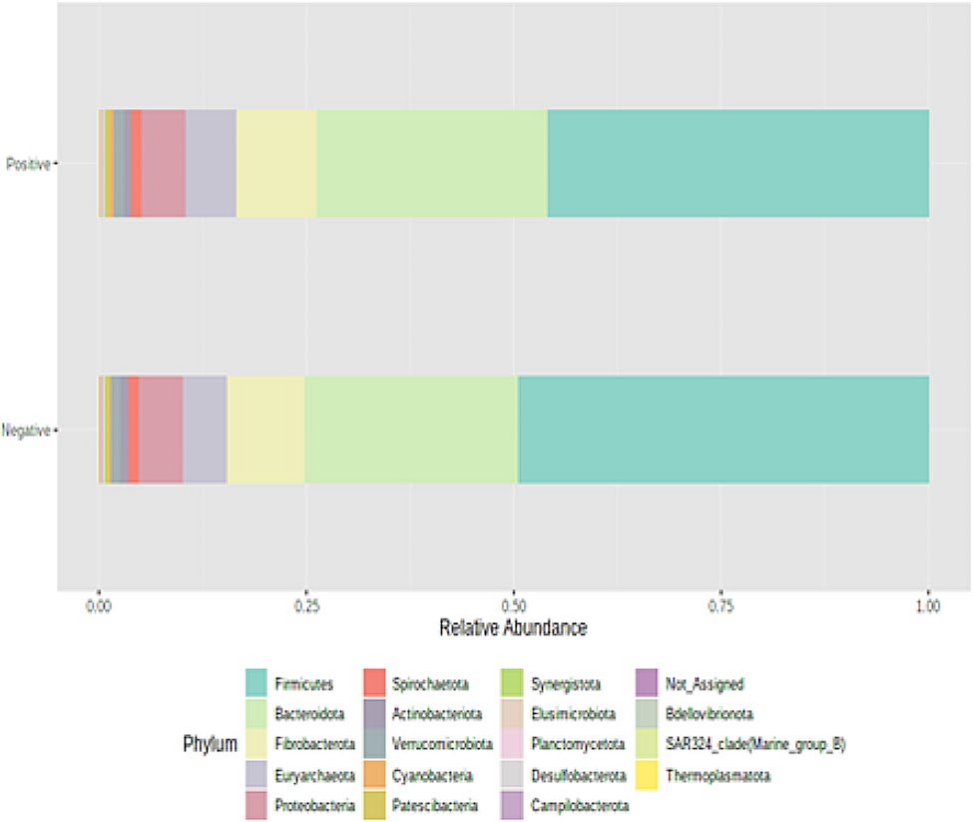
Relative abundance of rumen microbial taxa at the phylum level in beef steers with divergent residual feed intake phenotypes

**Fig. 2:**
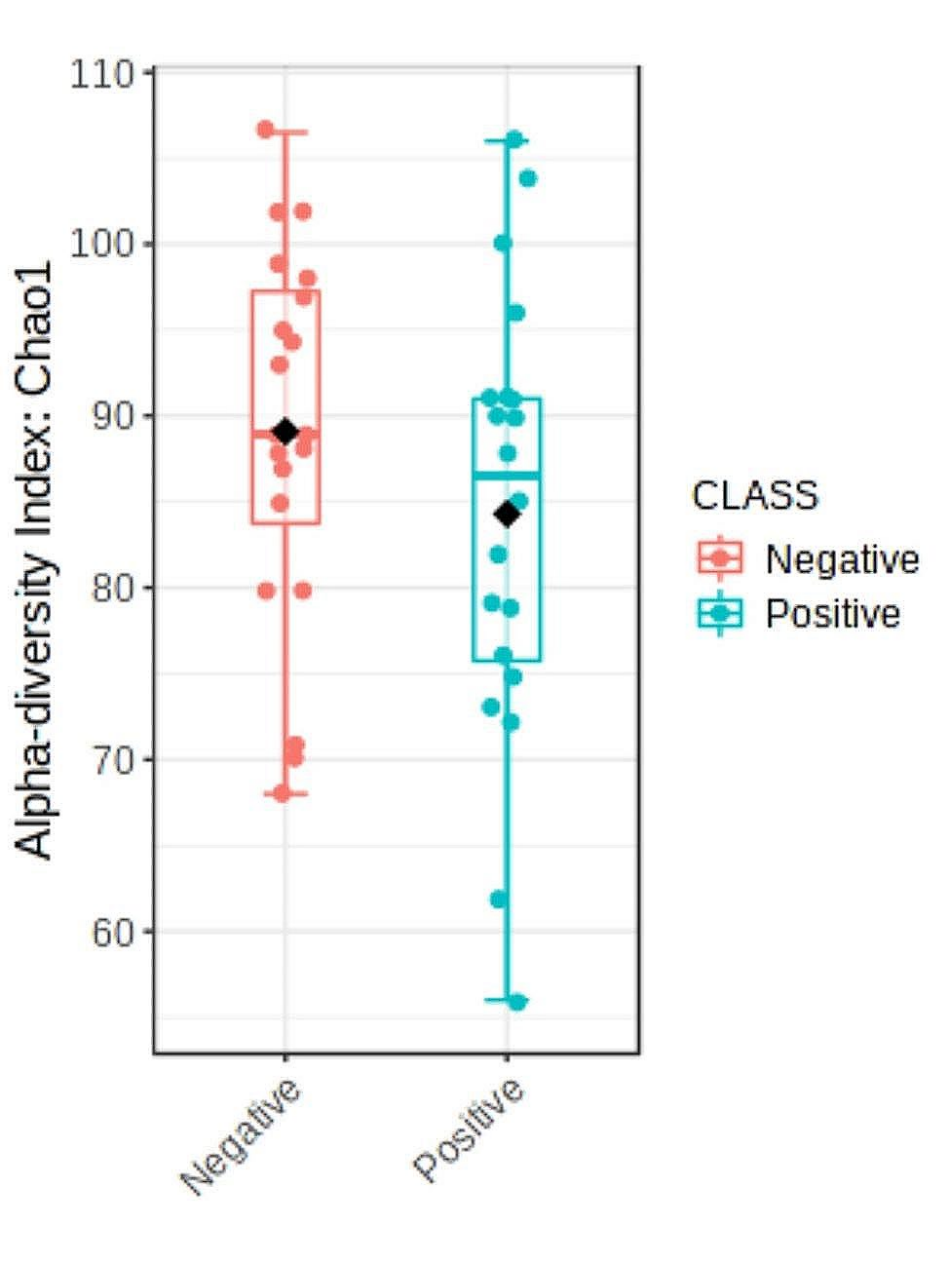
Alpha diversity index (Chao1) of rumen microbial taxa in beef steers with divergent residual feed intake phenotypes (*P*-value = 0.31)

**Fig. 3:**
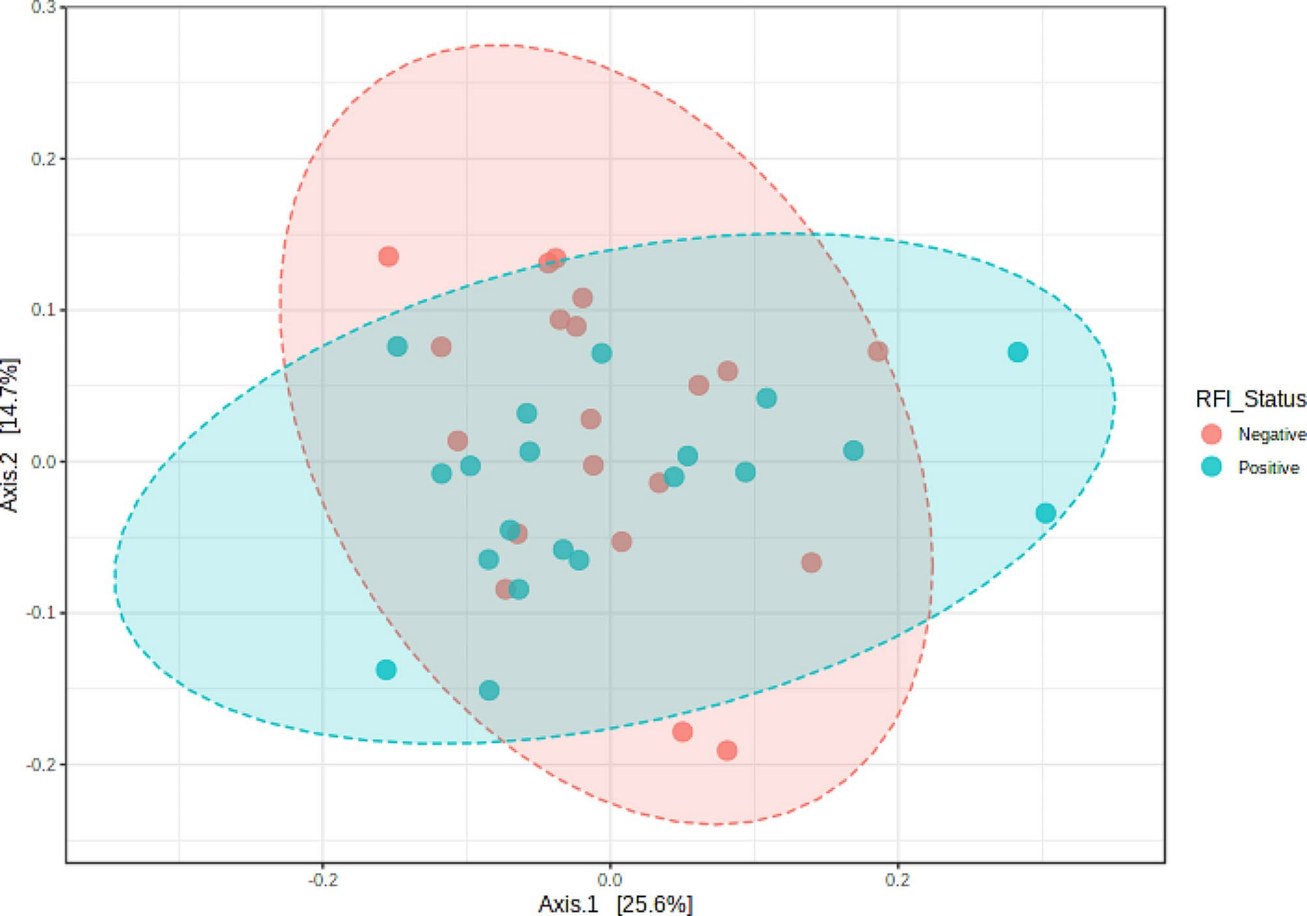
Principal coordinates analysis (PCoA) of ruminal microbiota based on an unweighted unifrac distance (Beta diversity *P* = 0.53)

**Fig. 4:**
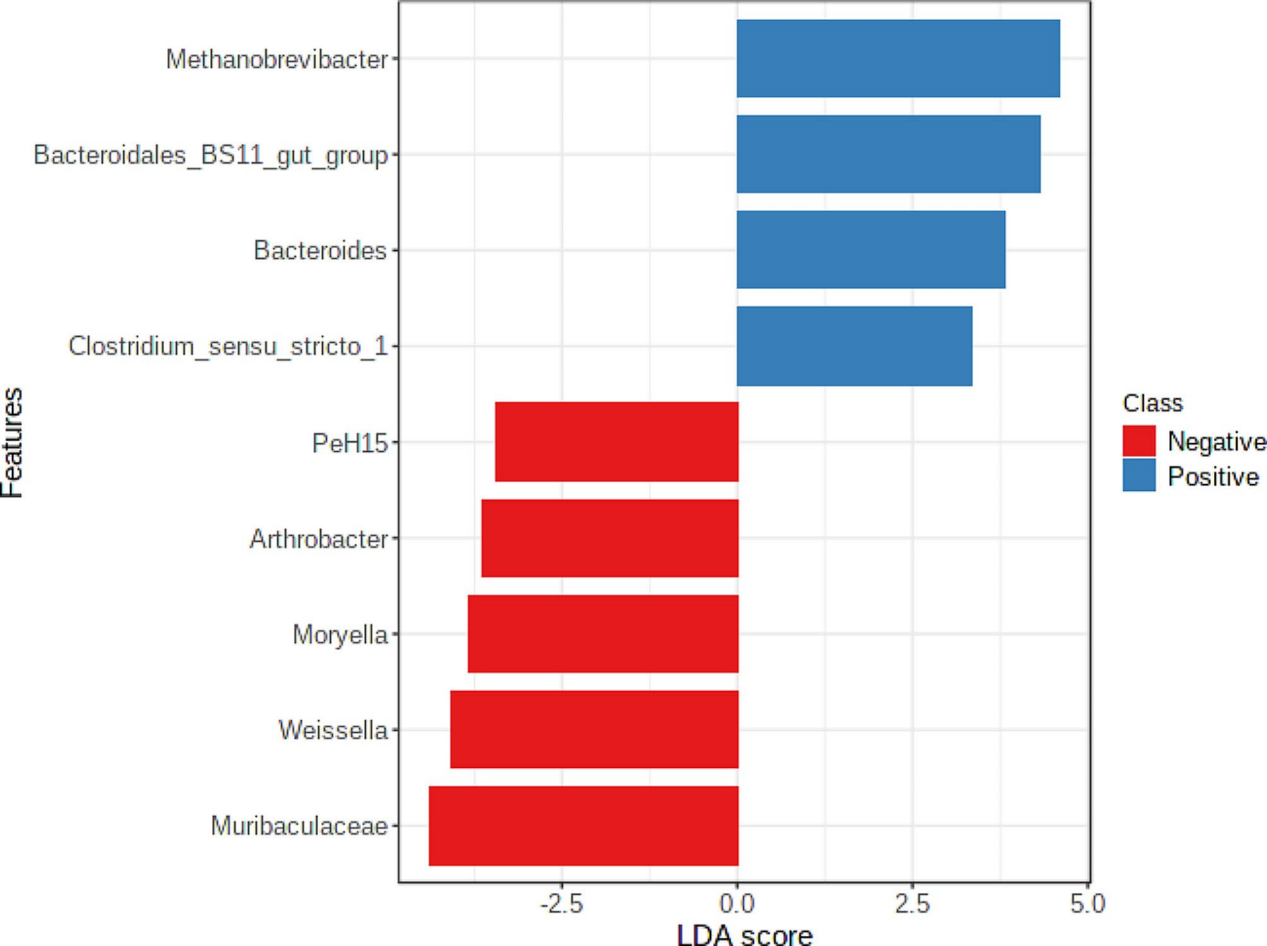
Linear discriminant analysis effect size (LEfSe) of rumen microbiota of beef steer with divergent residual feed intake phenotypes. The linear discriminant analysis plot indicates the most differentially abundant taxa found by ranking according to their effect size (≥ 2.0) at the genus

The high-throughput sequencing yielded approximately 166,378 ± 22,215 reads per sample. The rarefaction analysis revealed that the number of sequences utilized for all the samples was sufficient to ascertain the overall number of sequence types (Supplementary Fig. 1). To identify the differentially abundant taxa mostly affected between the two groups of steers, we compared the rumen microbial population using the metagenomic biomarker discovery approach, LEfSe. This method employs a nonparametric Wilcoxon sum-rank test, followed by linear discriminant analysis, to evaluate the effect size of each differentially abundant taxon. At the phylum level, the microbial community composition of the rumen samples was predominantly composed of Bacteroidota and Firmicutes (Fig. 1). There was no difference in alpha (Fig. 2; *P* = 0.31; Chao 1 index) or beta (Fig. 3; *P* = 0.53; Bray-Curtis distance analysis) diversity indices between the two groups of beef steers. Likewise, no differences were observed between the two groups of beef steers at the phylum level. At the genus level, the relative abundance of *PeH15, Arthrobacter, Moryella, Weissella* and *Muribaculaceae* were enriched in low-RFI steers, while *Methanobrevibacter, Bacteroidales_BS11_gut_group, Bacteroides* and *Clostridium_sensu_stricto_1* were reduced (Fig. 4). The relative abundance of *Clostridium sensu stricto 1, Bacteroides*, *Bacteriodales_BS11_gut_group* were reduced in the low-RFI steers while those of *Weissella, PeH15*, *Arthrobacter Muribaculaceae and Moryella* were greater compared to the high-RFI steers (Fig. 4).

### Whole-blood and hepatic immune gene expression

**Fig. 5:**
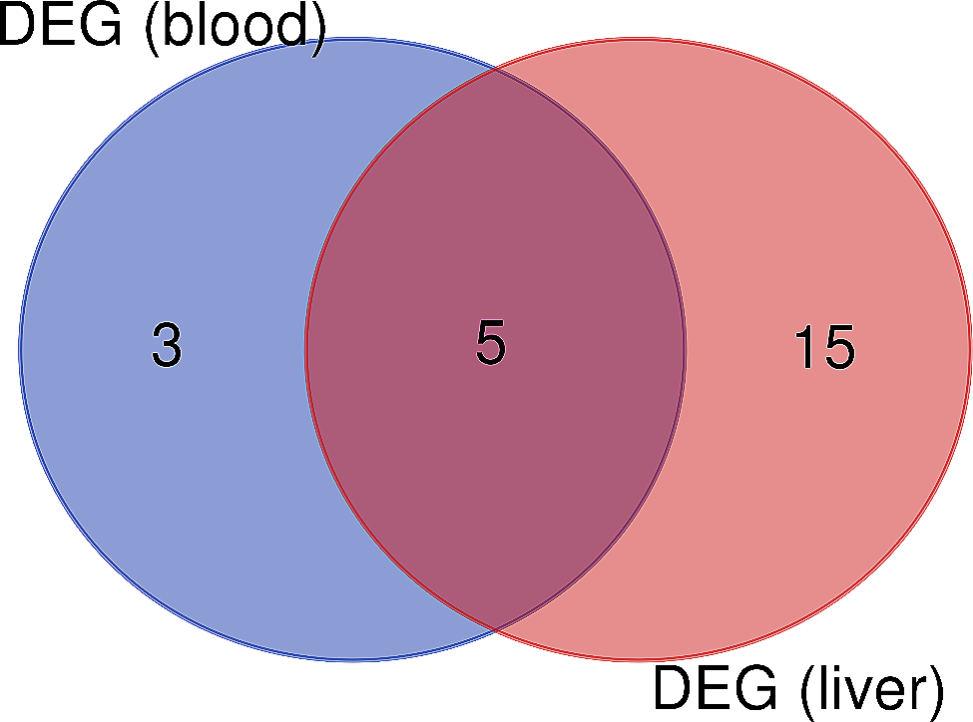
Differentially expressed whole blood and liver innate and adaptive immune genes in low- compared with high-RFI steers. The overlapping region of the diagram represents the differentially expressed genes (IL17A, CXCL10, MPO, IL2, and LY96) detected in both the whole blood and liver of low-RFI steers

To assess the differential expression of both innate and adaptive immune genes between the low-RFI and high-RFI steers, we utilized 84 gene array panel for transcriptome analysis. The transcript abundance of these genes in the blood and liver are shown in Supplementary Tables 1 and 2, respectively. The genes having *P*-value ≤ 0.05 and FC ≥ 1.5 or 2.0 in the blood or liver, respectively, were considered differentially expressed as presented in Tables 2 and 3. Comparing differential gene expression between low-RFI and high-RFI steers, out of the 84 genes analyzed, only eight were significantly upregulated in the blood (Table 2) and twenty in the liver of low-RFI steers (Table 3). Interestingly, five of these differentially expressed genes (IL17A, CXCL10, MPO, IL2 and LY96) had overlapping expression in both the blood and liver (Fig. 5).

**Table 2:** Fold change of whole blood innate and adaptive immune genes expression in low- compared with high-RFI steers^1^

Gene symbol	Gene name	FC^2^
CSF2	Colony stimulating factor 2 (granulocyte-macrophage)	24.22
IL17A	Interleukin 17 A	19.13
IL2	Interleukin 2	3.86
MBL2	Mannose-binding lectin (protein C) 2, soluble	2.21
MPO	Myeloperoxidase	1.91
LY96	Lymphocyte antigen 96	1.89
CXCL10	Chemokine (C-X-C motif) ligand 10	1.75
IL15	Interleukin 15	1.60

^1^Low-RFI = feed-efficient beef steers, high-RFI = feed inefficient beef steers

^2^Fold change (FC; relative to high-RFI)

**Table 3:** Fold change of hepatic innate and adaptive immune genes expression in low- compared with high-RFI steers^1^

Gene symbol	Gene name	FC^2^
IL2	Interleukin 2	36.08
IFNB1	Interferon, beta 1, fibroblast	23.09
TNF	Tumor necrosis factor	22.67
CXCL8	Interleukin 8	11.53
CASP1	Caspase 1, apoptosis-related cysteine peptidase (interleukin 1, beta, convertase)	5.88
IL4	Interleukin 4	5.48
CD40LG	CD40 ligand	5.02
IL17A	Interleukin 17 A	4.82
MX1	Myxovirus (influenza virus) resistance 1, interferon-inducible protein p78 (mouse)	4.67
TLR5	Toll-like receptor 5	4.65
MPO	Myeloperoxidase	4.46
IL13	Interleukin 13	4.36
CXCL10	Chemokine (C-X-C motif) ligand 10	4.36
LYZ	Lysozyme	4.10
IFNG	Interferon, gamma	4.00
LY96	Lymphocyte antigen 96	3.51
STAT4	Signal transducer and activator of transcription 4	3.49
TBX21	T-box 21	3.46
IL6	Interleukin 6 (interferon, beta 2)	3.39
GATA3	GATA binding protein 3	3.35

^1^Low-RFI = feed-efficient beef steers, high-RFI = feed inefficient beef steers

^2^Fold change (FC; relative to high-RFI)

### Gene ontology and functional pathways

Functional analysis, pathway and GO enrichment of the DE genes revealed top 15 or 20 most significant pathways in whole blood or liver respectively (Tables 4 and 5). From the whole blood transcriptome gene set, the topmost enriched pathways are directly related to pattern recognition receptor signaling, positive regulation of tumor necrosis factor production, macrophage activation and differentiation, and positive regulation of interleukin-10 production among others (Table 4). Of interest, LY96, IL2, IL15 and IL17A were most common in several pathways. While in the liver, most significantly enriched pathways include positive regulation of immunoglobulin production, positive regulation of interleukin-13 production, vascular endothelial growth factor production, and regulation of complement-dependent cytotoxicity (Table 5). Additionally, we found IL2, CSF2 and IL17A, some of the most upregulated genes in the blood of low-RFI to be connected to the production and regulation of interleukin-17 and interleukin-23 production pathways.

**Table 4:** Gene ontology showing enriched biological processes, molecular functions, and cellular components of whole blood innate and adaptive immune genes in low- compared with high-RFI steers^1^

	Gene(s)	Raw *P*-value	FDR^2^
**GO biological process**			
Detection of lipopolysaccharide	LY96	3.54E-06	1.70E-03
Positive regulation of interleukin-23 production	CSF2, IL17A	4.55E-06	2.12E-03
Positive regulation of interleukin-17 production	IL2, IL15	2.65E-05	7.34E-03
Macrophage differentiation	IL15, CSF2	2.65E-05	7.20E-03
Toll-like receptor 4 signaling pathway	LY96	3.19E-05	8.36E-03
Positive regulation of tyrosine phosphorylation of STAT protein	IL2, IL15, CSF2	6.79E-07	6.52E-04
Receptor signaling pathway via JAK-STAT	IL15, CSF2	7.06E-05	1.43E-02
Defense response to fungus	MPO, IL17A	8.37E-05	1.59E-02
Positive regulation of tumor necrosis factor production	LY96, IL17A	2.01E-06	1.16E-03
Pattern recognition receptor signaling pathway	LY96, MBL2	2.58E-06	1.38E-03
Positive regulation of phagocytosis	IL15, MBL2	2.00E-04	3.31E-02
**GO molecular function**			
Toll-like receptor 4 binding	LY96	1.90E-06	1.42E-03
Lipopolysaccharide immune receptor activity	LY96	3.54E-06	1.99E-03
Pattern recognition receptor activity	LY96, MBL-2	2.91E-05	1.31E-02
**GO cellular component**			
Lipopolysaccharide receptor complex	LY96	4.55E-06	2.85E-03

^1^Low-RFI = feed-efficient beef steers, high-RFI = feed inefficient beef steers

^2^False discovery rate (FDR; relative to high-RFI)

**Table 5:** Gene ontology showing enriched biological processes, molecular functions, and cellular components of hepatic innate and adaptive immune genes in low- compared with high-RFI steers^1^

	Gene (s)	Raw *P*-value	FDR^2^
**GO biological process**			
Positive regulation of immunoglobulin production	IL13, IL6, TBX21, IL4, IL	1.23E-09	2.42E-07
Positive regulation of interleukin-10 production	IL13, IL6, CD40LG, IL4,	3.75E-08	5.62E-06
T-helper cell differentiation	GATA3, IL6, TBX21, IL4	5.39E-08	7.68E-06
Macrophage activation	IL13, IFNG, IL4, TNF	9.23E-08	1.19E-05
Positive regulation of interleukin-13 production	GATA3, IL4	1.67E-07	1.96E-05
Vascular endothelial growth factor production	TNF, IL6, IFN	2.22E-07	2.46E-05
Microglial cell activation	IFNG, IL4, TNF	5.63E-07	5.55E-05
Positive regulation of cytokine production involved in inflammatory response	IL6, TNF, IL17A	2.30E-06	1.80E-04
Regulation of acute inflammatory response	IL6, IL4, TNF	2.92E-06	2.19E-04
Regulation of complement-dependent cytotoxicity	IL13, IL4	6.31E-06	4.07E-04
Wnt signaling pathway involved in kidney development	GATA3	1.05E-05	6.22E-04
Positive regulation of vitamin metabolic process	IFNG, TNF	1.05E-05	6.36E-04
Detection of lipopolysaccharide	LY96	2.94E-05	1.50E-03
Positive regulation of mast cell activation involved in immune response	IFNG, TNF	6.91E-05	2.96E-03
Positive regulation of interleukin-23 production	GATA3	3.78E-05	1.82E-03
Positive regulation of interleukin-5 production	GATA3	3.78E-05	1.82E-03
Positive regulation of isotype switching to IgG isotypes	TBX21, IL4	4.72E-05	2.17E-03
**GO molecular function**			
Tumor necrosis factor receptor binding	TNF, CD40LG,	3.51E-10	1.97E-07
CD40 receptor binding	CD40LG	3.54E-08	1.59E-05
CXCR chemokine receptor binding	CXCL8, CXCL10	9.73E-07	3.65E-04
Interleukin-8 receptor binding	CXCL8,	6.31E-06	2.18E-03
Toll-like receptor 4 binding	LY96	1.58E-05	4.73E-03
Interleukin-2 receptor binding	GATA3, IL2	2.20E-05	5.83E-03
Lipopolysaccharide immune receptor activity	LY96	2.94E-05	7.34E-03
Toll-like receptor binding	LY96	9.51E-05	2.14E-02
**GO cellular component**			
Lipopolysaccharide receptor complex	LY96	3.78E-05	1.77E-02

^1^Low-RFI = feed-efficient beef steers, high-RFI = feed inefficient beef steers

^2^False discovery rate (FDR; relative to high-RFI)

## Discussion

This study determined the rumen microbiome and immune gene expression profile of beef steers with divergent RFI using 16 S rRNA gene sequencing and targeted transcriptome analyses, respectively. The results of our study revealed a lower relative abundance of *Methanobrevibacter*, a genus of archaea that belongs to the *Methanobacteriaceae* family, in low-RFI compared to high-RFI beef steers. This might imply that the low-RFI steers could partition their methane production via alternative pathways especially when a lower proportion of H_2_ and CO_2_ is being produced during the fermentation process by the rest of microbiota. For instance, carbohydrates are fermented to propionic acid with no net loss of CO_2_ and thus lower substrate for *Methanobrevibacter* to produce methane [32, 33].Previous studies have shown that *Methanobrevibacter* are predominant methanogens in the rumen and their abundance has also been correlated with higher levels of methane emissions [34, 35] and poorer feed efficiency [36, 37]. Cattle with negative RFI phenotype have been reported to have reduced daily methane production [7]. In addition, we noted that the relative abundance of *Muribaculaceae and Moryella* were greater in Low-RFI beef cattle. *Muribaculaceae* is a family of bacteria that produces enzymes capable of degrading complex carbohydrates and has been reported to produce short-chain fatty acids [38, 39], which play important roles in regulating immune function and energy metabolism. A recent study revealed that the abundance of *Muribaculaceae* in the rumen is positively correlated with feed efficiency and other production traits such as milk components [40] and negatively correlated with methane production in Holstein dairy cows [41].

As seen in our result, the relative abundance of *Moryella* was greater and that *Clostridium_sensu_stricto_1* was lower in low-RFI compared to high-RFI. Previous studies have shown that species of *Moryella* play a key role in the breakdown of complex carbohydrates and the production of volatile fatty acids (VFAs) such as acetate, propionate, and butyrate, which are important energy sources that support improved health and performance of ruminants [42, 43]. A study correlating individual RFI values with bacterial abundances in feces reported that *Clostridium* I is associated with high RFI in chickens [5, 44]. In fact, an overgrowth of *Clostridium sensu stricto 1* was reported to be associated with necrotic enteritis in human subjects, consequently depicting unhealthy microbiota [45, 46]. Therefore, the lower relative abundance of *Clostridium_sensu_stricto_1* in low-RFI beef steers might suggest a robust and healthy microbiome which might translate to better use of nutrients.

Of outmost importance, we provide the first evidence of increased relative abundance of *Weissella, PeH15* and *Arthrobacter* in the low-RFI steers. These genera have been identified as probiotics with immune-boosting potential in humans, fish and chicken [47]. Probiotics in ruminants influence enzyme production leading to efficient digestion of nutrients, improved growth and performance and robust immunity [29, 48, 49]. In this sense, greater relative abundance of *Weissella, PeH15* and *Arthrobacter* in the rumen of low-RFI group suggest a possible role in activation and initialization of immunomodulatory properties, improved growth, and feed efficiency enhancement. Immune response is related to cascades of metabolic processes and require high metabolic demands. This is also largely connected to the probiotic activities of rumen microbiome. Due to the energy cost associated with immune system activation, immune competence is suggested to be one of the major physiological processes that contributes to variation in RFI in Angus beef cattle [50, 51]. Our study showed that certain innate immune genes such as LY96, TLR4 and MBL-2 which play a significant role in detection of lipopolysaccharide, pattern recognition receptor, microphage differentiation and positive regulation of phagocytosis were found upregulated in the blood and liver of low-RFI beef steers with divergent RFI phenotypes. This is important in the initial pathogen recognition and subsequent activation of downstream immune signaling pathways that recruit the adaptive immune response. In addition, toll-like receptors (TLRs), nod-like receptors (NLRs), scavenger receptors, and C-type lectin receptors are pattern-recognition receptors that play a vital role in maintaining pathogen specificity and consequent protection against microbial invasion [52, 53]. Therefore, the enrichment of pathways including pattern recognition receptor, lipopolysaccharide-mediated signaling pathway, microphage differentiation and positive regulation of phagocytosis in our study suggest that low-RFI steers possess a better mechanism for pathogen recognition, reduction of endotoxin and other bacterial products both in the systemic circulation and the hepatocytes. In fact, observed upregulation in expression levels of LY96, TLR4 and MBL-2 and their associated pathways in the liver of low-RFI animals is reasonable because the liver is constantly exposed to gut-derived bacterial products and endotoxins through its main blood supply, the portal vein and is rich in Kupffer cells which helps in detoxification of endotoxins leading to increased concentration of circulating endotoxin with consequent systemic inflammation [54, 55]. This immunological imbalance impairs efficient partitioning of nutrients leaving livestock in poor condition of growth and performance [56].

Pathways such as positive regulation of cytokine production involved in inflammatory response and vascular endothelial growth factor production were enriched, and vital pro-inflammatory cytokines such as Tumor Necrosis Factor (TNF-α), CASP1 (interlukine-1 convertase), interlukine-6 (IL-6), C-X-C motif chemokine ligand 10 (CXCL10) and interferon beta and gamma (IFN-β /γ) were found to be differentially upregulated both in the blood and liver of low-RFI beef steers. Interestingly, we also found that interferon (IFN-β /γ), interferon gamma-induced protein 10 (CXCL10 or IP-10) and their associated pathways were enriched in low-RFI compared to high-RFI beef steers. Taken together, our results indicated that the upregulated pro-inflammatory cytokines coupled with other innate immune genes mediate complex signaling cascade of events in low-RFI beef steers towards recognizing, binding, and marking of pathogens for destruction while maintaining cellular homeostasis. These further suggest a robust innate immune system in low-RFI steers, capable of initiating a prompt response against foreign entities compared to high-RFI steers.

The GO terms associated with positive regulation of immunoglobulin production, T cell differentiation involved in immune response, and positive regulation of vitamin metabolic process were the most overrepresented pathways for differentially co-expressed genes such as GATA3, IL6, TBX21, IL4 and MEF2C including the enrichment of alpha-beta T cell differentiation in the liver of low-RFI beef steers. These pathways might suggest that the animals possess a robust adaptive immune mechanism for balancing both the catabolic-and anabolic-immune pathways despite their low dry matter intake.

Overall, we showed a significant correlation between the microbial community, immune response and divergent RFI phenotypes. Mostly, dietary nutrients are partitioned towards the immune related processes rather than being used for growth and thus reduces animal’s feed efficiency. This is extremely relevant for immune-metabolic axis in livestock [57–59]. Therefore, the upregulation of our immune genes set and the enriched pathways in both the blood and liver of the low-RFI beef steers suggest that low-RFI beef steers possess a mechanism that allows for a prompt response to pathogen or any other foreign substances and consequently showcase a robust repertoire of both innate- and adaptive-immunity compared to high-RFI beef steers. While our study provides valuable insights into differential gene expression and associated gene ontology patterns, it is crucial to exercise caution when generalizing the biological implications drawn from our GO analyses, especially given the constraints imposed by the low number of DEGs. Future studies with larger sample sizes or additional validation experiments would allow for more comprehensive interpretations of the gene ontology results.

## Conclusion

In summary, our study demonstrates that low-RFI beef cattle possess a robust and efficient immune response to inflammation, characterized by the upregulation of genes involved in pathogen recognition, intracellular signaling, activation of antimicrobial mechanisms, and phagocytotic killing. These animals exhibit a superior ability to quickly eliminate pathogens and effectively compared to their high-RFI counterparts. Additionally, the relative abundance of *Methanobrevibacter* was lower in low-RFI beef steers, which was probably associated with a reduced methane production. The increased abundance of *Weissella, PeH15*, and *Arthrobacter* in low-RFI steers suggests a potential role of these taxa in the rumen microbiome in initiating immunomodulatory properties, improved growth, and feed efficiency. Future studies utilizing larger cohorts of steers are needed to further investigate the functional characterization of rumen microbes that may be important for the immune system efficiency and nutrient-harvesting in ruminants.

### Electronic supplementary material

Below is the link to the electronic supplementary material.

Supplementary Material 1

Supplementary Material 2

Supplementary Material 3

## Data Availability

The datasets analyzed in this study are all available in NCBI (BioProject number PRJNA955175).
