# Supplementary material for: Characterization of rumen microbiome and immune genes expression of crossbred beef steers with divergent residual feed intake phenotypes
Source: BMC Genomics. 2024 Mar 5;25:245. doi: 10.1186/s12864-024-10150-3 (PMC10913640; doi:10.1186/s12864-024-10150-3)
Supplement: Supplementary file 1 — Supplementary Material 1 [file 12864_2024_10150_MOESM1_ESM.pdf]

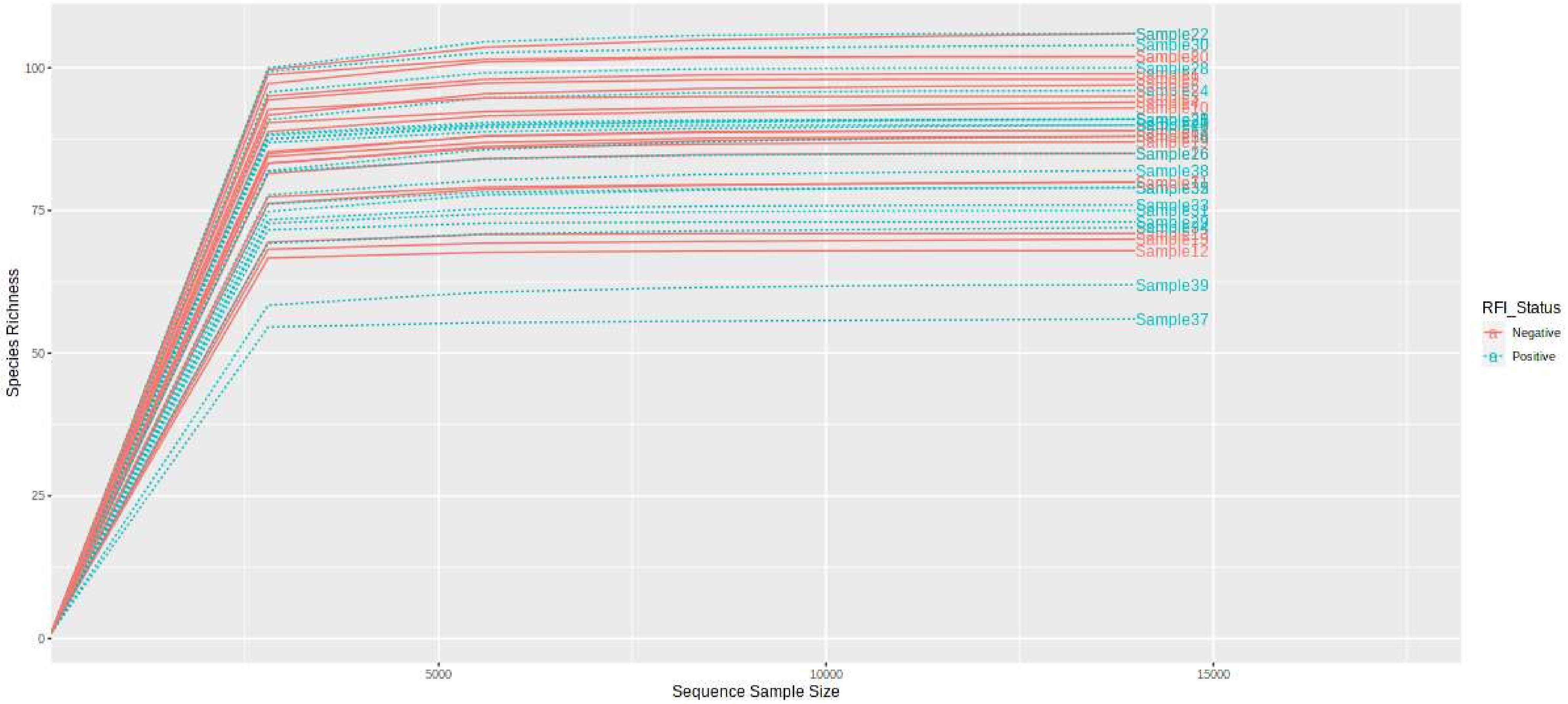

Supplementary Figure S1: Rarefaction curve for sequences from RFI negative (red) and RFI positive (green) samples
