# Supplementary material for: Characterization of rumen microbiome and immune genes expression of crossbred beef steers with divergent residual feed intake phenotypes
Source: BMC Genomics. 2024 Mar 5;25:245. doi: 10.1186/s12864-024-10150-3 (PMC10913640; doi:10.1186/s12864-024-10150-3)
Supplement: Supplementary file 2 — Supplementary Material 2 [file 12864_2024_10150_MOESM2_ESM.docx]

Table S1. Whole blood immune gene expressions in beef steers divergent for high or low RFI.

| Gene symbol | Gene name | FC | *P*-value |
| --- | --- | --- | --- |
| CSF2 | Colony stimulating factor 2 (granulocyte-macrophage) | 24.22 | 0.01 |
| IL17A | Interleukin 17A | 19.13 | 0.03 |
| IL2 | Interleukin 2 | 3.86 | 0.02 |
| MBL2 | Mannose-binding lectin (protein C) 2, soluble | 2.21 | 0.04 |
| MPO | Myeloperoxidase | 1.91 | 0.02 |
| LY96 | Lymphocyte antigen 96 | 1.89 | 0.02 |
| CXCL10 | Chemokine (C-X-C motif) ligand 10 | 1.75 | 0.03 |
| IL15 | Interleukin 15 | 1.60 | 0.02 |
| DDX58 | DEAD (Asp-Glu-Ala-Asp) box polypeptide 58 | 1.56 | 0.39 |
| IL1B | Interleukin 1, beta | 1.50 | 0.08 |
| TLR4 | Toll-like receptor 4 | 1.47 | 0.12 |
| NOD2 | Nucleotide-binding oligomerization domain containing 2 | 1.46 | 0.06 |
| IFNB1 | Interferon, beta 1, fibroblast | 1.44 | 0.18 |
| LOC512672 | Major histocompatibility complex, class I | 1.41 | 0.20 |
| IL18 | Interleukin 18 (interferon-gamma-inducing factor) | 1.39 | 0.22 |
| SLC11A1 | Solute carrier family 11 (proton-coupled divalent metal ion transporters), member 1 | 1.38 | 0.17 |
| TLR8 | Toll-like receptor 8 | 1.37 | 0.14 |
| STAT3 | Signal transducer and activator of transcription 3 (acute-phase response factor) | 1.36 | 0.17 |
| TLR5 | Toll-like receptor 5 | 1.36 | 0.46 |
| CD86 | CD86 molecule | 1.35 | 0.18 |
| IRF3 | Interferon regulatory factor 3 | 1.34 | 0.07 |
| FASLG | Fas ligand | 1.34 | 0.44 |
| NFKB1 | Nuclear factor of kappa light polypeptide gene enhancer in B-cells 1 | 1.33 | 0.08 |
| MYD88 | Myeloid differentiation primary response gene (88) | 1.32 | 0.24 |
| TLR2 | Toll-like receptor 2 | 1.30 | 0.28 |
| TLR3 | Toll-like receptor 3 | 1.29 | 0.45 |
| FAS | Fas (TNF receptor superfamily, member 6) | 1.28 | 0.24 |
| CD40 | CD40 molecule, TNF receptor superfamily member 5 | 1.27 | 0.27 |
| IFNGR1 | Interferon gamma receptor 1 | 1.26 | 0.26 |
| CD4 | CD4 molecule | 1.24 | 0.19 |
| MX1 | Myxovirus (influenza virus) resistance 1, interferon-inducible protein p78 (mouse) | 1.22 | 0.81 |
| CASP1 | Caspase 1, apoptosis-related cysteine peptidase (interleukin 1, beta, convertase) | 1.21 | 0.43 |
| LYZ | Lysozyme | 1.21 | 0.61 |
| TLR9 | Toll-like receptor 9 | 1.21 | 0.97 |
| CD14 | CD14 molecule | 1.20 | 0.54 |
| CXCL8 | Interleukin 8 | 1.20 | 0.55 |
| TRAF6 | TNF receptor-associated factor 6 | 1.19 | 0.12 |
| NFKBIA | Nuclear factor of kappa light polypeptide gene enhancer in B-cells inhibitor, alpha | 1.18 | 0.24 |
| STAT4 | Signal transducer and activator of transcription 4 | 1.18 | 0.52 |
| NLRP3 | NLR family, pyrin domain containing 3 | 1.17 | 0.28 |
| IL23A | Interleukin 23, alpha subunit p19 | 1.16 | 0.42 |
| IFNAR1 | Interferon (alpha, beta and omega) receptor 1 | 1.14 | 0.29 |
| C3 | Complement component 3 | 1.13 | 0.52 |
| CD80 | CD80 molecule | 1.11 | 0.39 |
| STAT6 | Signal transducer and activator of transcription 6, interleukin-4 induced | 1.11 | 0.77 |
| CCL5 | Chemokine (C-C motif) ligand 5 | 1.11 | 0.82 |
| CD40LG | CD40 ligand | 1.10 | 0.76 |
| BOLA-A | Major histocompatibility complex, class I, A -A | 1.09 | 0.77 |
| TYK2 | Tyrosine kinase 2 | 1.08 | 0.45 |
| JAK2 | Janus kinase 2 | 1.08 | 0.86 |
| MAPK1 | Mitogen-activated protein kinase 1 | 1.07 | 0.76 |
| IL1R1 | Interleukin 1 receptor, type I | 1.07 | 0.88 |
| IRAK1 | Interleukin-1 receptor-associated kinase 1 | 1.06 | 0.93 |
| TNF | Tumor necrosis factor | 1.04 | 0.72 |
| LOC616942 | Major histocompatibility complex, class I, A-like | 1.04 | 0.95 |
| BOLA | MHC class I heavy chain | 1.01 | 0.61 |
| TLR1 | Toll-like receptor 1 | 1.01 | 0.87 |
| RORC | RAR-related orphan receptor C | 1.00 | 0.83 |
| CCL2 | Chemokine (C-C motif) ligand 2 | 1.00 | 0.97 |
| CXCR3 | Chemokine (C-X-C motif) receptor 3 | 0.97 | 0.73 |
| ITGAM | Integrin, alpha M (complement component 3 receptor 3 subunit) | 0.97 | 0.76 |
| TBX21 | T-box 21 | 0.97 | 0.85 |
| TLR6 | Toll-like receptor 6 | 0.96 | 0.60 |
| CD8A | CD8a molecule | 0.95 | 0.64 |
| STAT1 | Signal transducer and activator of transcription 1, 91kDa | 0.94 | 0.62 |
| CCR6 | Chemokine (C-C motif) receptor 6 | 0.94 | 0.75 |
| GATA3 | GATA binding protein 3 | 0.92 | 0.70 |
| FOXP3 | Forkhead box P3 | 0.91 | 0.79 |
| CCR4 | Chemokine (C-C motif) receptor 4 | 0.89 | 0.61 |
| IRF7 | Interferon regulatory factor 7 | 0.88 | 0.68 |
| APCS | Amyloid P component, serum | 0.85 | 0.37 |
| MAPK3 | Mitogen-activated protein kinase 3 | 0.83 | 0.23 |
| TICAM1 | Toll-like receptor adaptor molecule 1 | 0.82 | 0.72 |
| CCR5 | Chemokine (C-C motif) receptor 5 | 0.81 | 0.18 |
| ICAM1 | Intercellular adhesion molecule 1 | 0.81 | 0.33 |
| TLR7 | Toll-like receptor 7 | 0.80 | 0.61 |
| IL4 | Interleukin 4 | 0.77 | 0.26 |
| CCR8 | Chemokine (C-C motif) receptor 8 | 0.75 | 0.16 |
| IFNG | Interferon, gamma | 0.73 | 0.34 |
| IL6 | Interleukin 6 (interferon, beta 2) | 0.69 | 0.81 |
| IL1A | Interleukin 1, alpha | 0.53 | 0.33 |
| IL10 | Interleukin 10 | 0.42 | 0.33 |
| IL13 | Interleukin 13 | 0.29 | 0.21 |
| CRP | C-reactive protein, pentraxin-related | 0.24 | 0.34 |
